# The Preparation of *Drosophila* Embryos for Live-Imaging Using the Hanging Drop Protocol

**DOI:** 10.3791/1206

**Published:** 2009-03-13

**Authors:** Bruce H. Reed, Stephanie C. McMillan, Roopali Chaudhary

**Affiliations:** Department of Biology, University of Waterloo

## Abstract

Green fluorescent protein (GFP)-based timelapse live-imaging is a powerful technique for studying the genetic regulation of dynamic processes such as tissue morphogenesis, cell-cell adhesion, or cell death.  Drosophila embryos expressing GFP are readily imaged using either stereoscopic or confocal microscopy.  A goal of any live-imaging protocol is to minimize detrimental effects such as dehydration and hypoxia.  Previous protocols for preparing Drosophila embryos for live-imaging analysis have involved placing dechorionated embryos in halocarbon oil and sandwiching them between a halocarbon gas-permeable membrane and a coverslip^1-3^.  The introduction of compression through mounting embryos in this manner represents an undesirable complication for any biomechanical-based analysis of morphogenesis.  Our method, which we call the hanging drop protocol, results in excellent viability of embryos during live imaging and does not require that embryos be compressed.  Briefly, the hanging drop protocol involves the placement of embryos in a drop of halocarbon oil that is suspended from a coverslip, which is, in turn, fixed in position over a humid chamber.  In addition to providing gas exchange and preventing dehydration, this arrangement takes advantage of the buoyancy of embryos in halocarbon oil to prevent them from drifting out of position during timelapse acquisition. This video describes in detail how to collect and prepare Drosophila embryos for live imaging using the hanging drop protocol.  This protocol is suitable for imaging dechorionated embryos using stereomicroscopy or any upright compound fluorescence microscope.

**Figure:**
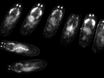


## Protocol

### Preparation

Collect embryos on standard grape-juice agar plates^4^.  It is convenient to use an automated Drosophila Egg Collector (Flymax Scientific Equipment Ltd.). Using synchronous staged embryo collections will reduce the number of dechorionations that must be performed in order to obtain adequate numbers of embryos at the desired developmental stage.
Prepare a dechorionation slide by attaching a piece of double-sided tape to a microscope slide; remove the backing from the tape.
Prepare the live imaging chamber by cutting a piece of tissue to fit in the well of the chamber.  Place the tissue in the well and wet it with distilled water.
Mix halocarbon oil (Halocarbon Products Corp.) viscosity series 700 and series 56 at a ratio of 1:1.  The mixture can be stored and used indefinitely.
Place several small drops of halocarbon oil on the surface of a coverslip (22 X 40 mm, No. 2).  The volume is not critical but should be in the order of 20-40 µl.

### Procedure

With the aid of a stereomicroscope, collect embryos from the grape juice-agar plates using either a pair of jeweller’s forceps (No. 5) or a very fine paint brush.  Embryos tend to stick to each other and to the forceps’ tips; they are easily gathered in clumps.
With the aid of a stereomicroscope, gently lower the clumps of embryos that have adhered to the tips of the forceps onto the surface of the tape on the dechorionation slide.
Again using the stereomicroscope, gently nudge or stroke the embryos with the side of the forceps in order to break open the outer waxy chorion without rupturing the inner vitelline membrane (the embryo will burst if the vitelline membrane is ruptured).
Once the outer chorion has been ruptured, tease the embryo from the chorion; the dechorionated embryo tends to adhere to the surface of the forceps.  Take care to avoid touching the dechorionated embryo to the surface of the tape.
As soon as an embryo is dechorionated, quickly transfer it to the previously prepared drop of halocarbon oil on the coverslip.  Touch the tip of the forceps carrying the dechorionated embryo to the surface of the halocarbon oil drop - this dislodges the embryo from the forceps into the oil.
Confirm transfer of the embryo to the oil drop.  This can be done with the naked eye provided that you work over a black background and use a fibre-optic illumination source set at an oblique angle.
Before attempting to dechorionate another embryo, clean any oil from the forceps.  Forceps coated in oil have less purchase on the chorion surface, making dechorionation more difficult.
Dechorionated embryos are buoyant in the halocarbon oil.  Using forceps or a paintbrush and while viewing through a stereomicroscope, push the embryo to the bottom of the oil drop and arrange it in the desired position.
Quickly invert the coverslip over the well of the live imaging chamber.  Confirm that the embryos are resting against the coverslip in the desired orientation.  Due to their bouyancy in the oil, the embryos will now float against the lower surface of the coverslip.  Fix the coverslip to the live imaging chamber with tape. Ventilate the chamber by cutting holes in the tape in the area where the tape spans the gap between the end of the coverslip and the end of the well of the live imaging chamber.
An upright fluorescence microscope or a fluorescence stereomicroscope can now be used to image the embryos.

## Discussion

We describe a new method of preparing Drosophila embryos for live imaging analysis, which we call the hanging drop protocol. Unfortunately, it is not possible to use the hanging drop protocol if working with an inverted microscope. In this case the sandwiching technique (as described in the abstract above) must be used and compression of the embryos remains a concern.

In experiments where cell shape and size are measured in order to calculate forces associated with morphogenetic movement, the use of an upright microscope and the hanging drop protocol is preferable owing to reduced embryo compression.  Also, reduced embryo compression using the hanging drop protocol has the consequence of presenting the round, undistorted surface of the embryo whereas the sandwiching technique presents a flattened surface.  When using confocal microscopy it is, therefore, necessary to collect a broader Z-stack (more slices per stack) when using the hanging drop protocol versus the sandwiching method.  The increased number of slices per Z-stack, however, increases acquisition time as well as any photo-toxicity or photo-bleaching associated with laser excitation.  Clearly, the experimental benefit associated with reduced compression is somewhat offset by an increase in Z-stack acquisition times.  Despite this increase in Z-stack acquisition time, however, we have observed excellent viability of embryos using the hanging drop protocol.

The hanging drop protocol is also limited to the observation of embryos by fluorescence microscopy, in which the light path of incident and emitted light does not pass through the live-imaging chamber. Live-imaging embryos using DIC microscopy or other non-fluorescence optics could be achieved by modifying the live-imaging chamber to allow a light path from the condenser through the suspended embryo.

A concern when using the hanging drop technique may be undesirable movement or “drifting” of embryos in the field of view during timelapse acquisition.  When floating against the underside of the inverted coverslip, we find that the embryos are remarkably stable and do not shift in position when using either dry or oil immersion objectives.  Multipoint timelapse acquisition using a motorized microscope stage also does not disrupt the positions of embryos that are prepared using the hanging drop protocol.  Any movement of embryos prepared using the hanging drop technique can be eliminated by reducing the size of the halocarbon oil drop and ensuring that separate oil drops are not fusing or wicking along the edge of the live-imaging chamber’s well.
